# Update on squamous cell carcinoma of the head and neck: ASCO annual meeting 2017

**DOI:** 10.1007/s12254-017-0358-9

**Published:** 2017-10-20

**Authors:** Teresa Magnes, Alexander Egle, Richard Greil, Thomas Melchardt

**Affiliations:** 1 Third Medical Department with Hematology and Medical Oncology, Paracelsus Medical University Salzburg, Müllner Hauptstraße 48, 5020 Salzburg, Austria; 2 Salzburg Cancer Research Institute, Salzburg, Austria; 3 Cancer Cluster Salzburg, Salzburg, Austria

**Keywords:** IDO1, Immunotherapy, Epacadostat, Cisplatin, Afatinib

## Abstract

At the annual ASCO meeting clinically relevant data concerning the management of advanced head and neck cancer that will influence clinical practice in the future were presented.

Chemoradiation with high-dose cisplatin remains the mainstay of treatment for patients with locally advanced squamous cell head and neck cancer. Adjuvant therapy with afatinib following chemoradiation failed to show clinical benefit. The combination of bevacizumab with platinum-based chemotherapy improved progression-free survival but did not lead to a significant difference in overall survival compared to chemotherapy alone. However, the addition of immunotherapy may improve multimodal treatment concepts in locally advanced disease and new treatment combinations might overcome resistance to checkpoint inhibition.

## Introduction

Squamous cell carcinoma of the head and neck (SCCHN) belongs to one of the most common tumor types worldwide and accounts for more than 550,000 new cases and 380,000 deaths per year [1]. Apart from the commonly known risk factors such as smoking tobacco products and alcohol consumption, infection with human papilloma virus (HPV) has become more and more important in the epidemiology and prognosis of SCCHN [2]. Most patients are diagnosed with locally advanced disease that often requires multimodal treatment approaches and the collaboration of radiation and medical oncologists as well as head and neck surgeons. Despite improvement of radiation and surgical techniques and the addition of chemotherapy and monoclonal antibodies in advanced disease more than half of all patients experience relapse of disease not suitable for treatment options with curative intention [3].

Since the publication of a phase III clinical trial by Vermorken et al. in 2008, the combination of cetuximab, cis- or carboplatin and 5‑fluorouracil has been the standard of care for patients with recurrent or metastatic SCCHN [4]. More recently, the PD-1 (Programmed cell death protein 1) antibody nivolumab and pembrolizumab have been approved for the treatment of patients with platinum-resistant disease. However, survival of patients with advanced head and neck cancer remains low and the response rates to checkpoint inhibition between 13–18% leave much room for improvement [5, 6].

At the ASCO annual meeting several clinical trials were presented in the field of locally advanced as well as recurrent and metastatic SCCHN.

## Locally advanced head and neck cancer

The current standard of care for patients with locally advanced head and neck cancer is tumor resection followed by platinum-based chemoradiation. If surgery is not feasible, definitive radiotherapy in combination with platinum chemotherapy or cetuximab is carried out with curative intention. A number of abstracts discussed this year in Chicago were aimed at the improvement of outcomes after adjuvant or definitive chemoradiation either by adapting systemic therapy during radiotherapy or by the addition of neoadjuvant or adjuvant treatment.

The LUX-Head & Neck 2 study was a phase 3 study investigating the addition of afatinib after chemoradiation in patients with locally advanced SCCHN. Afatinib, an oral tyrosine kinase inhibitor, irreversibly blocks signal transmission from the epidermal growth factor receptor (EGFR) and human epidermal growth factor receptor (HER) 2, 3, and 4. Afatinib modestly improved the progression-free survival (PFS) when compared to methotrexate in patients with recurrent or metastatic disease [7]. In the LUX-2 trial patients with no evidence of disease after chemoradiation were randomized between afatinib and placebo for up to 18 months. The study was closed early due to the unlikelihood of achieving a significant survival benefit after interim analysis and afatinib showed no benefit in disease-free survival (DFS) independent of PTEN expression, HPV status and the number of smoking pack years [8].

In another trial presented by Dr. Noronha from India 300 patients receiving postoperative or definitive chemoradiation for locally advanced SCCHN were randomized between a cisplatin dose of 100 mg/m^2^ every 3 weeks and 30 mg/m^2^ weekly. The majority of patients had surgery before adjuvant chemoradiation and showed high-risk features such as perinodal extension. The locoregional relapse rate after two years was significantly higher in the weekly cisplatin arm compared to the 3‑weekly arm. There was a trend towards improved DFS of patients receiving high-dose cisplatin every three weeks but the study did not have enough statistical power to show survival differences. The rate of grade 3 or higher adverse events was significantly higher in the 3‑weekly cisplatin arm and this mainly came from an increased number of hematologic toxicities in this group. Interestingly, the incidence of renal toxicity was very low in both arms of the study and the low median age of 47 years in the 3‑weekly cisplatin arm may implicate that these findings are not representative for European patients [9].

Two studies that were discussed during the immunotherapy for head and neck session at ASCO annual meeting 2017 investigated the addition of pembrolizumab to multimodal treatment in patients with locally advanced SCCHN. Dr. Powell presented the safety data of the combination of pembrolizumab with definitive chemoradiation. Patients received weekly cisplatin at a dose of 40 mg/m^2^, 200 mg pembrolizumab every three weeks and radiation at 2 Gy daily for 35 planned fractions. At the time of the interim analysis presented at the meeting 27 patients were enrolled. Out of these patients, 21 (78%) received all planned doses of pembrolizumab. The study was discontinued on three patients due to immune-related adverse events and on three more due to protocol reasons. The majority of patients received at least a cumulative dose of 200 mg/m^2^ cisplatin and all patients completed radiotherapy as planned per protocol without significant treatment delays. One patient’s death occurred after the completion of chemoradiation due to gastrointestinal bleeding in a patient who rejected intervention. At day 150 after the start of chemoradiation with pembrolizumab, 21 patients (78%) had a complete response, 4 patients had a partial response and the tumor of one patient had progressed. The expansion cohorts of the study now include 34 patients with HPV-positive and 23 patients with HPV-negative disease and further efficacy analyses are awaited [10].

In a phase II trial presented by Dr. Uppaluri patients with stage III or IV locally advanced, HPV-negative SCCHN received one dose of 200 mg pembrolizumab prior to tumor resection. Those patients with extracapsular extension or positive margins on pathologic exam were treated with chemoradiation with cisplatin followed by pembrolizumab. There were no serious safety concerns and no unexpected delays of tumor resection. None of the 14 patients with a survival follow-up of at least 12 months experienced locoregional recurrence or disease specific death. Pathologic treatment response defined by “tumor necrosis and/or giant cell/histiocytic reaction to keratinous debris in more than 10% of the tumor area” was achieved in 42% of the patients. Furthermore, only 10 out of 24 patients who received a single dose of pembrolizumab before surgery showed high-risk features on pathologic exam compared to an institutional historic rate of 71%, which was seen as a hint for an early response to pembrolizumab [11].

## Recurrent or metastatic head and neck cancer

Several studies in patients with recurrent or metastatic SCCHN were presented during the oral sessions and poster presentations at this year’s ASCO annual meeting. Dr. Argiris discussed the results of a phase III trial where bevacizumab was added to platinum-based chemotherapy. The study was designed before the data on cetuximab in combination with platinum-based chemotherapy were presented in 2008. Despite improvement of PFS and response rate, the primary endpoint of improving overall survival (OS) was not reached by the addition of bevacizumab. Furthermore, a higher rate of serious bleeding events was seen in the bevacizumab arm [12].

In the Keynote-12 study, which led to approval of pembrolizumab for head and neck cancer in the United States of America, the overall response rate was 18% [6]. Due to this low response rate many efforts are taken to identify patients with a higher probability of response to immunotherapy. Dr. Haddad now presented data from whole exome sequencing and gene expression profiling (GEP) in this study population. Results show that the T‑cell inflamed GEP was associated with response in HPV/Epstein-Bar-virus-positive and -negative disease while the mutational load only predicted response in viral negative patients [13].

Dr. Hamid discussed preliminary data of epacadostat plus pembrolizumab in patients with SCCHN. Epacadostat is an oral inhibitor of IDO1, an intracellular enzyme that leads to immunosuppression. In the phase 2 part of the trial, patients with recurrent or metastatic SCCHN with at least one prior chemotherapy including a platinum compound were treated with 100 mg epacadostat daily and 200 mg pembrolizumab every 3 weeks. The overall response rate and disease control rate were 34% and 39%, respectively. Responses were seen independent of PD-1 and HPV status and most responses were ongoing. Grade 3 or 4 adverse events were reported in 18% of the patients including one pneumonitis. One patient died due to aspiration pneumonia. However, pneumonitis could not be ruled out [14].

**Fig. 1:**
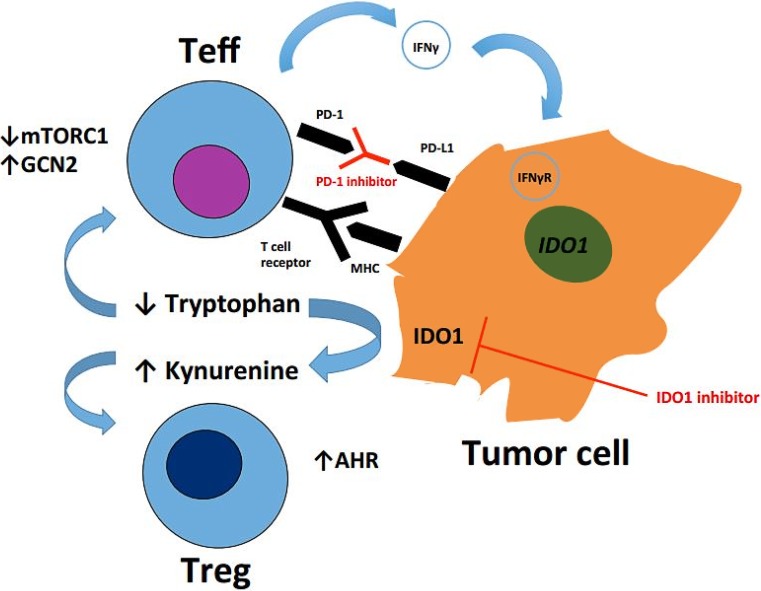
Epacadostat as an oral inhibitor of IDO1 may overcome the resistance to immunotherapy in advanced head and neck cancer. IDO1 enzyme induces an immunosuppressive state by increasing kynurenine and reducing tryoptophan levels. PD-1 inhibition increases IDO1 expression in tumor cells and both PD-L1 and IDO1 are regulated by interferon gamma. *Teff* T effector cell, *Treg* T regulatory cell, *mTOR* mechanistic target of rapamycin, *GCN* general control nonderepressible, *PD-1* programmed cell death protein 1, *PD-L1* programmed cell death protein ligand 1, *IFNγ* interferon gamma, *IFNγR* interferon gamma receptor, *IDO* indoleamine deoxygenase, *MHC* major histocompatibility complex, *AHR* aryl hydrocarbon receptor. (Adapted from [15])

The authors concluded that such combinations may improve the benefit of checkpoint inhibitors in the future (Fig. 1).

## Conclusion

The data presented at the ASCO annual meeting 2017 in the field of head and neck cancer are encouraging and will influence clinical practice in upcoming years. For now, chemoradiation with cisplatin every 3 weeks remains the standard of care in eligible patients with locally advanced SCCHN. Afatinib failed to show improvement of DFS when added as adjuvant treatment after chemoradiation [8]. However, the studies on the addition of checkpoint inhibition to surgery and/or chemoradiation are promising and randomized trials further investigating the clinical benefit are ongoing. In recurrent or metastatic SCCHN, the current treatment standards were not changed by the data presented at this year’s ASCO annual meeting. Platinum-based chemotherapy with cetuximab remains the standard of care as first-line therapy in platinum-sensitive disease and checkpoint inhibitors are approved in patients with platinum-resistant disease. Due to the failure to show an OS benefit and also the safety concerns bevacizumab will not become a standard in SCCHN. The addition of epacadostat to pembrolizumab showed that new treatment combinations might overcome resistance to checkpoint inhibition in some patients leading to an increase in the ORR when compared to anti-PD-1 monotherapy in earlier studies but longer follow-up and randomized trials are needed for a final evaluation.

### Take home message

At the annual ASCO meeting clinically relevant data concerning the management of advanced head and neck cancer that will influence clinical practice in the future were presented. The addition of immunotherapy may improve multimodal treatment concepts in locally advanced disease and new treatment combinations might overcome resistance to checkpoint inhibition.

## Abbreviations

DFS: Disease-free survival
EGFR: Epidermal growth factor receptor
GEP: Gene expression profiling
HER: Human epidermal growth factor receptor
HPV: Human papilloma virus
OS: Overall survival
PFS: Progression-free survival
SCCHN: Squamous cell carcinoma of the head and neck
